# Gingival biotype modification with collagen matrix or autogenous subepithelial connective tissue graft: Histologic and volumetric analyses in a beagle model

**DOI:** 10.1016/j.heliyon.2023.e15026

**Published:** 2023-04-11

**Authors:** Yoonsub Lee, Jung-Tae Lee, Hee-seung Han, Seunghan Oh, Young-Dan Cho, Sungtae Kim

**Affiliations:** a Department of Periodontology, School of Dentistry and Dental Research Institute, Seoul National University and Seoul National University Dental Hospital, Seoul, South Korea; b Department of Periodontics, One-Stop Specialty Center, Seoul National University, Dental Hospital, Seoul 05698, South Korea; c Department of Dental Biomaterials, The Institute of Biomaterial and Implant, School of Dentistry, Wonkwang University, Iksan, South Korea

**Keywords:** Collagen, Connective tissue, Gingival recession, Periodontal biotype, VEGF, Periodontology, Biologic width

## Abstract

**Objectives:**

To evaluate the volumetric effect and biocompatibility of porcine tendon-derived type I collagen matrix graft (CG) in gingival biotype modification (GBM) compared with subepithelial connective tissue graft (SCTG) in a beagle model.

**Methods:**

Surface analysis using scanning electron microscopy and a collagen degradation assay of CG was performed *in vitro*. Six adult dogs were used in *in vivo* experiment, and each received autologous SCTG or CG at the anterior side. Histometric and three-dimensional digital volume analyses were conducted to compare quantitative changes in CG and SCTG in GBM. Immunohistochemical analysis was performed for the qualitative evaluation of CG compared to SCTG.

**Results:**

CG had a double-layered structure, and its degradation was slower than that of other well-reported materials. No critical problems were associated with the healing procedure. Changes in gingival thickness and volume in the CG and SCTG groups were equivalent, with no significant differences between the groups. Type I collagen and vascular endothelial growth factor expression levels were similar in both groups.

**Significance:**

CG and SCTG had equivalent potential for GBM in terms of quantity and quality. Additionally, CG could be used as a reasonable substitute for SCTG, making surgery convenient and predicting successful clinical outcomes.

## Highlights

• Porcine tendon derived type I collagen matrix graft (CG) was proposed that it could replace sub-epithelial connective tissue graft (SCTG) in gingival biotype modification (GBM).
• The change of gingival thickness and volume of CG group was equivalent to SCTG group with similar expression levels of type I collagen and VEGF.
• CG may be proposed as a reasonable substitute for SCTG in GBM, which makes surgery convenient and predict successful clinical outcomes.

## 1 Introduction

The term “gingival biotype” refers to the gingival thickness in the facio-palatal or facio-lingual dimension, while the “periodontal biotype” refers not only to the gingival thickness, but also to other features, including tooth shape, gingival contour, alveolar bone morphotype, and amount of keratinized gingiva [1]. Thin biotypes are more vulnerable to injury and likely to induce gingival recession than thick biotypes, because thin gingiva can have an effect on plaque-related inflammatory lesions, making it prone to tissue destruction [2].

Numerous applications, including non-surgical periodontal therapy, mucogingival therapy, guided tissue regeneration (GTR), and implant dentistry, have shown evidence of impact of gingival thickness. Patients with gingiva less than 1.5 mm thick experienced attachment loss after non-surgical periodontal therapy, whereas no attachment loss was seen at sites with gingiva over 2 mm thick [3]. A significant moderate correlation occurred between a critical gingival thickness threshold of >1.1 mm and weighted mean root coverage and weighted complete root coverage according to a systematic review and meta-analysis [4].

Thin gingival thickness of less than 2 mm is associated with slightly greater initial peri-implant bone loss around implants compared with thick gingival thickness for securing the supracrestal tissue attachment of the gingiva [[5], [6], [7], [8], [9]]. Thus, the presence of soft tissues with a thickness >2.0 mm around implants is crucial in preserving healthy peri-implant tissues and preventing alveolar bone loss through the biological protection [10].

**Fig. 1:**
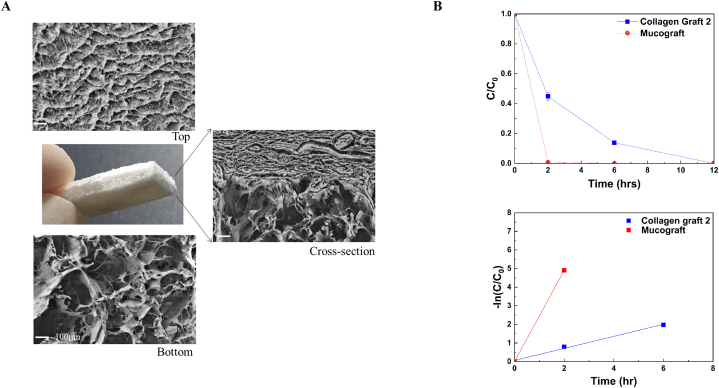
Characteristics of CG. (A) Microstructure of CG observed by FE-SEM. Scale bar = 100 μm, 100× magnification. (B) Degradation behavior and kinetic analysis of CG. Degradation behavior of experimental CG (top). Kinetic analysis of the degradation curves (bottom).

Based on these findings, surgical procedures for gingival biotype modification (GBM), such as gingival tissue augmentation [6], have been proposed to increase gingival thickness to maintain the biologic width and minimize alveolar bone loss (Fig. 1A). In GBM, subepithelial connective tissue graft (SCTG) is commonly recognized as goldstandard [11,12]. However, postoperative complications, such as uncontrolled bleeding, pain, and infection of the palatal donor site, limit its establishment as a routine procedure [[13], [14], [15], [16]]. The collagen matrix graft (CG) can be used in place of SCTG in gingival tissue augmentation, and several studies have shown similar results as SCTG in increasing gingival thickness [17,18] in preclinical studies.

In this study, we characterized CG material and evaluated the biocompatibility and volumetric effects of CG compared to SCTG on GBM in a beagle model using histometric and three-dimensional (3D) digital volumetric analysis.

## 2 Materials and methods

### 2.1 Materials

A commercially available CG (Collagen Graft 2®, Genoss, Suwon, Korea) with a double-layered structure was used (Fig. 1A); the upper layer is compact, while the lower layer is porous and consists of type I collagen from the porcine tendon. A commercially available collagen matrix (Mucograft®; Geistlich Biomaterials, Wolhusen, Switzerland) was used as a control group to compare the degradation behavior of CG.

### 2.2 Characterization of materials

The microstructure of CG was observed using field emission scanning electron microscopy (FE-SEM, Zeiss Gemini Sigma 500, ZEISS, Germany). The top, bottom, and cross-section of the collagen sponge sectioned by cutting were coated with Pt for 2 min using a Pt sputter coater and imaged using FE-SEM at 0.02–30 kV. The range of pore sizes of each region at the top and bottom was measured based on the SEM images.

### 2.3 Collagen degradation assay

A collagen degradation assay was conducted by assessing the degree of degradation of the membrane stored in collagenase solution at various loading periods [19]. We selected Mucograft® as the control group to compare the degradation behavior of CG. First, the initial weight of the membrane specimen (15 × 20 mm) was measured, and a collagenase solution (50 U/ml) was prepared by dissolving collagenase (C0130; Sigma, MO, USA) in 0.1 M Tris-HCl buffer (T2016–7.5; Biosesang Co Ltd., Seongnam, Korea) containing 0.005 M CaCl_2_ (C1016; Sigma, MO, USA). In a 15 ml tube, one piece of the membrane was placed with 5 ml of the collagenase solution. The tube was then placed in a shaking incubator (JSSI-100C; JS Research Inc., Gongju, Korea, Rotation speed: 100 rpm) at 37 °C. After 2, 6, 12, and 24 h of incubation, the collagenase solution supernatant was carefully removed from the tube. To wash the remaining membrane, distilled water (10 ml) was added to the tube, which was gently shaken for 30 s by hand, and the solution was removed from the tube. The washing process was repeated three times. Subsequently, the tube containing the remaining membrane was frozen at −80 °C in a deep freezer (NF-140SF; Nihon Freezer, Japan) and dried for more than 6 h in a lyophilizer (FD5510; Ilshin Biobase Co. Ltd., Dongducheon, Korea). After lyophilization, the final weight of the dried membranes was measured. The degradation behavior of the membrane was evaluated by calculating the ratio of the initial weight of the membrane to the final weight of the remaining membrane.

### 2.4 Experimental animals

Sample size calculation was conducted using previous study [17,18] assuming increase of gingival thickness (effect size) is 0.5 mm with power of 0.8, significant level of 0.05. The study involved six adult beagle dogs (average age, 13 months; average weight, 13 kg). The sample size was determined based on the 3Rs principles in animal research. All dogs were housed in cages under constant room temperature (22 ± 2 °C) and humidity (50 ± 10%). The protocol was approved by the Institutional Animal Care and Use Committee of CRONEX, Seoul, Korea (approval No. 202003001) according to the ARRIVE guidelines for preclinical studies [20].

### 2.5 In vivo study design

**Fig. 2:**
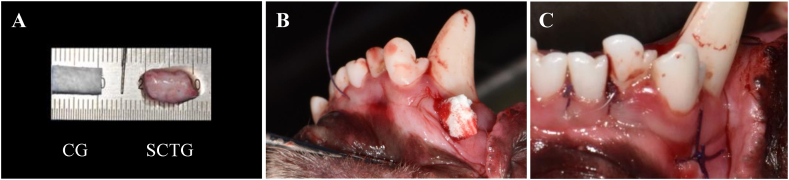
Experimental surgical procedure. (A) Sizes of both matched CG (left) and SCTG (right) from the palatal donor site (10 × 5 × 1.5–2 mm). (B) Insertion of CG into the subperiosteal tunnel. (C) Closing vertical incision using interrupted suture.

Twenty four sites on the labial side of the upper and lower anterior teeth were included as recipient sites (four sites per animal), which were then randomly allocated into two groups: SCTG and CG. Each dog received an autologous SCTG (width × height × depth = 10 × 5 × 1.5–2.0 mm) from their palatal donor site or porcine type I CG (10 × 5 × 1.5–2.0 mm) at the labial side of the second incisor (Fig. 2A). To evaluate and compare the quantitative effects of CG and SCTG on changes in gingival thickness or volume, dental cast models were fabricated at three time points: before surgery, and 1 and 5 months after surgery. Additionally, 3D digital volumetric analysis was performed. After 5 months, all animals were sacrificed, and samples were obtained for histometric and immunohistochemical (IHC) analyses. For each part (dimensional digital volumetric analysis, and histometric & IHC analysis), one expert conducted the analysis.

### 2.6 Surgical procedure

All dogs were anesthetized with a 1:1 mixture of Zoletil (0.1 mg/kg; Zoletil®50, Virbac S.A, France) and xylazine hydrochloride (0.1 mg/kg; Rompun, Bayer, Germany) intramuscularly before the surgery. Inhalation anesthesia was performed with 2% isoflurane (Isoflurane, Piramal Critical Care, United States) in 100% oxygen, and local anesthesia was administered by infiltration of 2% lidocaine hydrochloride and 1:100,000 epinephrine (Huons, Seongnam, Korea) at the donor and recipient sites. Prior to the experimental surgery, all dogs underwent scaling and plaque control. At the recipient sites, a vertical incision was made at the center of the second and third incisors on the buccal attached gingiva. Furthermore, a subperiosteal tunnel was formed mesially using a specifically designed elevator (CM9; Osung, Gimpo, Korea), allowing the graft to advance coronally to the marginal gingiva of the third incisor. At the donor site, SCTG was harvested from both sides of the palatal vault, and adipose tissue and epithelium were dissected from the graft (Fig. 2A). The donor site was treated with a hemostatic collagen matrix and continuous locking sutures were applied. Additionally, CG or SCTG was inserted into each subperiosteal tunnel (Fig. 2B), and an interrupted suture was performed (Fig. 2C). Surgical procedure was performed by one trained periodontist. After the surgery, subcutaneous administration of analgesics (carprofen, 5 mg/kg) and antibiotics (enrofloxacin, 0.2 ml/kg) was performed for 3 days to relieve pain and prevent infection. The surgical sites were treated with 0.2% chlorhexidine (Hexamedine; Bukwang Pharmaceutical, Seoul, Korea) daily for 10 days after surgery.

### 2.7 Histometric analyses

**Fig. 3:**
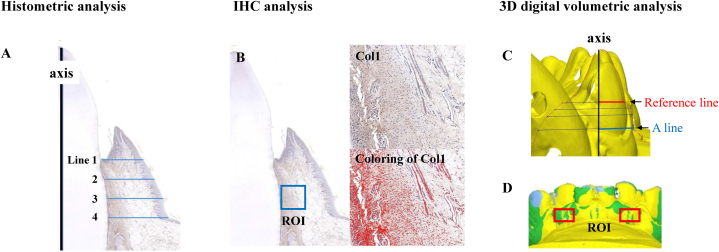
Histometric, immunohistochemical, and 3D digital volumetric analysis. (A) Exemplary image showing histometric analysis of gingival thickness (H&E, collagen type I immune staining). The black line indicates the tooth axis, and the blue lines indicate imaginary trisection lines perpendicular to the tooth axis, where the measurement of the soft tissue thickness was conducted. The line at the top (Line 1) was drawn from the base of the gingival sulcus to the outermost epithelium, perpendicular to the tooth axis. Then, the line at the bottom (Line 4) was drawn from MGJ to the root surface, perpendicular to the tooth axis. (B) Exemplary image showing histometric analysis of quantification for Col I and VEGF. The blue square indicates ROI (500 × 500 μm) for evaluating Col I and VEGF expression (Top) Representative image of Col I immunochemical staining in CG or SCTG (Bottom) The red section indicates where Col I staining is observed using the color threshold by Image J (20 × magnification). (C) Evaluation of gingival thickness change on the cross-section of the second incisor (black line: tooth axis, red line: reference line connecting the labial and lingual marginal gingiva, blue line (A-line): 2 mm apical to the baseline). (D) Superimposition of scanned data based on period to evaluate volume changes (yellow: before surgery, blue: 1 month after surgery, green: 5 months after the surgery, red box: ROI).

Five months after surgery, all dogs were euthanized using suxamethonium chloride hydrate (50 mg; Succipharm®, Komipharm, Gyeonggi, Korea). The resected specimens were fixed in 10% neutral buffered formalin. After dehydration and embedding in paraffin, 5.0-μm-thick serial sections were prepared. Three of the most central sections were selected: one stained with hematoxylin-eosin for histometric evaluation, and two for IHC. Histometric analysis was performed by two experienced researchers, YSL and HSH, and the measurements were conducted with image analysis software (Image-Pro Plus, Media Cybernetics, Silver Spring, MD). Within the two imaginary lines drawn perpendicular to the second incisor axis at the base of the junctional epithelium (Line 1, Fig. 3A) and mucogingival junction (MGJ) (Line 4, Fig. 3A), quaternary lines (Lines 2 and 3, Fig. 3A) were drawn within the soft tissue range. The thickness of the soft tissue, including the periodontal ligament, connective tissue, and epithelial tissue, was measured at quaternary lines, and statistical analysis (ANOVA, Kruskal–Wallis test) was conducted to analyze differences in soft tissue thickness in each group.

### 2.8 Immunohistochemical analysis

The sections were deparaffinized and hydrated, and antigen retrieval was performed using antigen retrieval buffer (Dako co., Glostrup, Denmark). Each section was incubated with the primary antibodies, anti-vascular endothelial growth factor (VEGF) (Novus, nb100-664), and anti-collagen I alpha (Novus, nbp1-77457) at room temperature for 1 h, followed by incubation with the secondary antibody (REAL Envision HRP Rabbit/Mouse Detection System, Dako co., Glostrup, Denmark) for 30 min. The sections were assessed using a digital slide scanner and computer software (PANNORAMIC 250 Flash III and Caseviewer, 3DHISTECH, Ltd. H-1141 Budapest, Öv u. 3., Hungary). For the IHC analysis, after drawing an imaginary line perpendicular to the second incisor axis at the base of the junctional epithelium and MGJ, a 500 × 500 μm square adjacent to the root surface was set as region of interest (ROI) (Fig. 3B). Subsequently, color thresholds were set using an image analysis program (Image J, National Institutes of Health, US) for the regions where type I collagen (Col I) and VEGF were observed, and Col I and VEGF expressions were quantified as the area ratio for each group.

### 2.9 Three-dimensional digital volumetric analysis

Impressions were taken before and 1 and 5 months after surgery using a polyvinyl siloxane impression material (Aquasil Ultra LV®, Dentsply, Konstanz, Germany), and a tray suitable for the oral structure of an adult dog was fabricated with a 3D scanner (3Shape, Copenhagen, Denmark, and exocad, exocad GmbH, Germany) and 3D printer (Asiga MX UV, Asiga, Australia). After fabrication of the study casts using a super hard dental stone (SNOW ROCK®, Bluewin, Gunpo, Korea), they were scanned using a dental scanner (ZEISS COMET 5 M, Oberkochen, Germany). Image data were then superimposed based on the time point using software (Geomagic Design X and Control X, 3DSYSTEMS, SC, USA), with the 1st incisor as the reference point. Lastly, a 3D digital volumetric analysis was performed to measure the changes in gingival thickness and volume. To evaluate changes in gingival thickness, a linear measurement using a cross-sectional method was performed [21,22]. In cross-sectionally superimposed images, imaginary lines on the second incisor connecting the central of buccal and lingual gingival margins were set as reference lines. Next, an A-line, which was perpendicular to the tooth axis, was drawn to the reference line at a 2.0 mm apical point (Fig. 3C). Then, changes in length in the A-line were evaluated at three time points: before and 1 and five months after surgery. For evaluation of volumetric change, superimposed images of the three time points were conducted using software (Geomagic Design X and Control X, 3DSYSTEMS, SC, USA). A rectangular area measuring 2.5 × 1.5 mm on the labial attached gingiva of the second incisor was set as the ROI and the amount of change between periods was calculated (Fig. 3D) [23].

### 2.10 Statistical analysis

Statistical analysis (ANOVA, Kruskal-Wallis test) was performed to analyze the differences in soft tissue thickness for each group. Additionally, the independent sample *t*-test and Mann–Whitney *U* test were used to compare the volume change between the SCTG and CG. Then, the paired sample *t*-test and Wilcoxon signed-rank test were used for comparison based on the period.

## 3 Results

### 3.1 Characterization of CG

SEM images showed the top and bottom views of CG (Fig. 1A). SEM image of the top view of CG showed small-pore-size structures, whereas the bottom view showed large-pore-size structures.

### 3.2 Collagen degradation assay

Fig. 1B shows the degradation behavior and kinetic analysis curves of the collagen membrane in collagenase solution. The degradation efficiency of the CG group was achieved at 55.05% after 2 h. In contrast, the degradation efficiency of the control group (Mucograft®) was 99.26% after 2 h, which was significantly higher than that of the CG group. Kinetic analysis of the degradation curves indicated that the degradation of the collagen membrane tested in this study followed a pseudo-first-order reaction [24].

### 3.3 Clinical findings

The postoperative healing process was uneventful and there were no inflammatory signs or other complications at the surgical site. All 6 dogs were included for histometric, 3D digital, and IHC analyses.

### 3.4 Histometric findings

Table 1A, Table 1B lists the results of the histometric analysis. The average change in gingival thickness was 1.80 ± 0.34 and 1.79 ± 0.40 mm in the SCTG and CG groups, respectively (Table 1A). The use of CG showed a similar increase in gingival thickness to SCTG. No significant differences were observed between the groups.

**Table 1A:** Histometric evaluation of gingival thickness (mm, Mean ± SD).

Group	Line 1	Line 2	Line 3	Line 4	Average of Line 1–4
SCTG	1.64 ± 0.29	1.79 ± 0.37	1.85 ± 0.36	1.94 ± 0.44	1.80 ± 0.34
CG	1.68 ± 0.28	1.78 ± 0.35	1.84 ± 0.47	1.87 ± 0.53	1.79 ± 0.40

**Table 1B:** Change of gingival thickness and volume on the 3D digital analysis (Mean ± SD).

	Group	Measurement	Pre-op.	Post op. 1 M	Post op. 5 M
**Thickness** (mm)	SCTG	A-line	3.27 ± 0.62	3.32 ± 0.46	3.33 ± 0.43
		Change	0	0.05 ± 0.45	0.06 ± 0.36
	CG	A-line	3.19 ± 0.62	3.26 ± 0.36	3.30 ± 0.44
		Change	0	0.07 ± 0.38	0.11 ± 0.32
**Volume** (mm^3^)	SCTG	Volume	8.57 ± 2.24	9.03 ± 2.30	9.05 ± 2.42
		Change	0	0.45 ± 1.07	0.48 ± 1.12
	CG	Volume	8.55 ± 1.38	9.24 ± 1.00*	9.25 ± 1.43*
		Change	0	0.69 ± 0.81	0.70 ± 0.81

SCTG, sub-epithelial connective tissue graft.

CG, collagen graft.

*p < 0.05.

### 3.5 3D digital analysis findings

Changes in gingival thickness and volume are summarized in Table 1B. A 3D digital cross-section analysis of the dental cast model (Fig. 3C) showed that after 1 month at A-line, the gingival thickness increased by 0.05 ± 0.45 mm and 0.07 ± 0.38 mm in the SCTG and CG groups, respectively. After 5 months, the increase in soft tissue thickness was 0.06 ± 0.36 mm and 0.11 ± 0.32 mm in the SCTG and CG groups, respectively (Table 1B). In the 3D digital volumetric analysis, the increase in soft tissue volume after 1 month was 0.45 ± 1.07 mm and 0.69 ± 0.81 mm in the SCTG and CG groups, respectively. After 5 months, the increase in soft tissue thickness was 0.48 ± 1.12 mm and 0.70 ± 0.81 mm in the SCTG and CG groups, respectively. The CG group at one (*p* = 0.023) and five months (*p* = 0.035) showed a significant increase in volume (Table 1B).

### 3.6 IHC findings

**Fig. 4:**
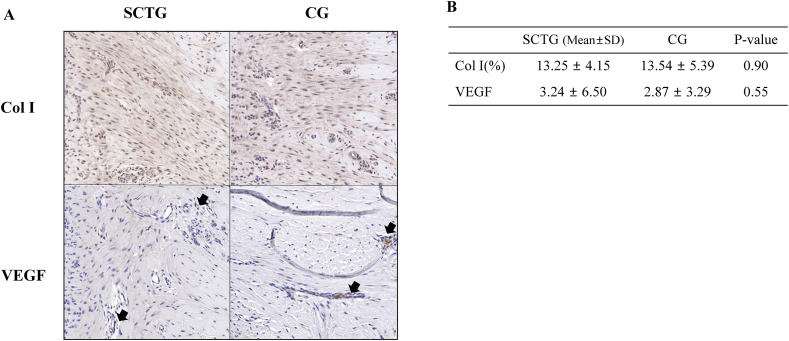
Comparison of Col I and VEGF expression. (A) Representative images of Col I and VEGF immunohistochemical staining (30× magnification). Col I was stained with a reddish color. The black arrows indicate the location of VEGF staining. (B) Quantification of Col I and VEGF expression by immunohistochemical staining (mean ± SD).

Expression patterns of anti-collagen I alpha and anti-VEGF in tissue sections were detected by IHC (Fig. 4A) and the expression level was quantified using the ROI area ratio (Fig. 4B). Col I expression levels were 13.25 ± 4.15% and 13.54 ± 5.39% in the SCTG and CG groups, respectively, with no significant difference (*p* = 0.90). Additionally, VEGF expression levels in the SCTG and CG groups were 3.24 ± 6.50% and 2.87 ± 3.29%, respectively, with no significant difference (*p* = 0.55).

## 4 Discussion

There are many techniques in the gingival augmentation procedure. Well known technique is coronally advanced flap [25]. However, coronally advanced flap operation is available only when the flap thickness is moderately thick [32]. Envelop or tunnel technique is preferred where gingiva is relatively thin [26,27]. Gingival thickness of the dog was the reason we chose tunnel technique.

We used histometric and 3D digital analyses to evaluate and compare the effects of SCTG and CG on increase in gingival thickness and volume. Histometric analysis showed a similar increase in gingival thickness (Table 1A), and the result was consistent with that of a 3D digital analysis (Table 1B). These results support previous studies showing that CG is not inferior to SCTG in gingival tissue augmentation [18,22,28,29]. Accurate and detailed evaluation of increased gingival thickness is important for gingival augmentation for esthetic improvement and securing gingival thickness >2.0 mm around dental implants to maintain gingival health and prevent early bone loss [[5], [6], [7], [8], [9]]. Therefore, the effects of GBM should be thoroughly reviewed [30]. Based on a dog experiment using a non-cross-linked CG and SCTG porcine, the maximum increase in gingival thickness after 10 months was 0.66 ± 0.29 mm and 0.79 ± 0.37 mm in the SCTG and CG groups, respectively [18]. However, an average increase in gingival thickness of 0.13 ± 0.26 mm in the CG group and 0.01 ± 0.26 mm in the SCTG group was reported [18]. An animal study in which immediate implant and soft tissue augmentation was performed using cross-linked CG reported a 0.52-mm increase in SCTG and 0.25-mm decrease in the CG group at the time point of sacrifice, and the author indicated that the underlying alveolar bone remodeling offsets the increase in gingival volume [22]. Subsequently, a human study using cross-linked CG grafts was placed around the implant sites was evaluated through 3D digital analysis 3 months after the operation, and a 0.175-mm increase in the CG group and a 0.51-mm in the SCTG group on the crest were reported. The previous study also reported 0.59 mm for the CG group and 0.94 mm for the SCTG group on buccal ROI, with no significant difference between the groups [28]. Other clinical studies about gingival augmentation using SCTG reported 0.13–0.37-mm increase in the gingival thickness in 2–3 months [28,31]. Hence, based on these results, it is stated that the effect of gingival augmentation in dogs is inferior to that in humans because of the relatively thin gingival thickness, narrow attached gingiva width of dogs, and difficulty in behavioral control causing worse results [32,33].

The type of CG (cross-linked or non-cross-linked) is also a factor to consider [34]. In another study, the cross-linked membrane showed the initiation of blood vessel invasion at 8 weeks, whereas the entire organization and biodegradation were observed at 4 weeks in the non-cross-linked membrane [35]. Most studies on gingival augmentation using non-cross-linked CG reported shrinkage of the soft tissue volume for up to 6 months [22,28,36,37]. In the present study, we used cross-linked CG, which was completely biodegraded and blended into the tissue at 5 months, as shown by the histological image (Fig. 4); there was no significant difference in gingival thickness and volume for up to 5 months between the groups (Table 1B). It is estimated that the difference in the results for each study was due to the difference in the CG used.

In our study, we observed Col I and VEGF expression in the histological sections (Fig. 4). Type I collagen is predominant in reparative connective tissues [38] and VEGF is best known angiogenic factor and is upregulated during the early wound healing phase [39]. In our study, Col I expression at 5 months after surgery was similar between groups (Fig. 4), which implies that augmented gingival tissue with CG is equivalent to that with SCTG with respect to tissue quality, as reported in a previous study [40]. VEGF expression was also observed, but there was no significant difference between the groups. Some studies have reported that VEGF may play an important role in vascularization during engraftment [40]. After SCTG is harvested from the palatal donor site, free gingival tissues are separated from the blood circulation, followed by the necrotic process, which is a known stimulatory factor for VEGF expression [41]. Based on these results, we believe that CG was well engrafted through a process similar to SCTG.

The present study had several limitations. First, this is a proof-of-principle study in a preclinical setting, which could be different from the clinical setting. Second, multiple time points of histologic analysis are needed to provide biological insight into the biodegradation and healing process of the recipient site. Another limitation of the study was that relatively few study animals were used, which may influence the results. Finally, the results should be interpreted carefully because of the small sample size; therefore, clinical trials with larger sample sizes are needed to draw conclusions. Within the limitations of this study, SCTG and CG seemed to have equivalent quantities of gingival augmentation, and quality of gingival augmentation. These results suggest that CG could be used as a substitute for SCTG, which makes surgery convenient and allows successful clinical outcomes.

## Ethical approval statement

This study was approved by the Institutional Animal Care and Use Committee of CRONEX, Seoul, Korea (approval No. 202003001) according to the ARRIVE guidelines for preclinical studies.

## Funding

This work was supported by a research grant from 10.13039/501100016149Seoul National University Dental Hospital [No. 03-2020-0044].Also, it was supported by 10.13039/501100003725National Research Foundation of Korea (NRF) grants funded by the 10.13039/501100014188Korean government (10.13039/501100003725MSIT) (No. 2020R1C1C1005830/2022M3A9F3082330).

## Availability of data and materials

All data generated by this study are included in this manuscript.
