# Supplementary material for: Gingival biotype modification with collagen matrix or autogenous subepithelial connective tissue graft: Histologic and volumetric analyses in a beagle model
Source: Heliyon. 2023 Apr 11;9(4):e15026. doi: 10.1016/j.heliyon.2023.e15026 (PMC10161361; doi:10.1016/j.heliyon.2023.e15026)
Supplement: Multimedia component 1 [file mmc1.docx]

# **Gingival biotype modification with collagen matrix or autogenous subepithelial connective tissue graft: Histologic and volumetric analyses in a beagle model**

Yoonsub Lee1, Jung-Tae Lee^2^, Hee-seung Han^1^, Seunghan Oh^3^, Young-Dan Cho^1^, Sungtae Kim^1^

**Appendix Figures**


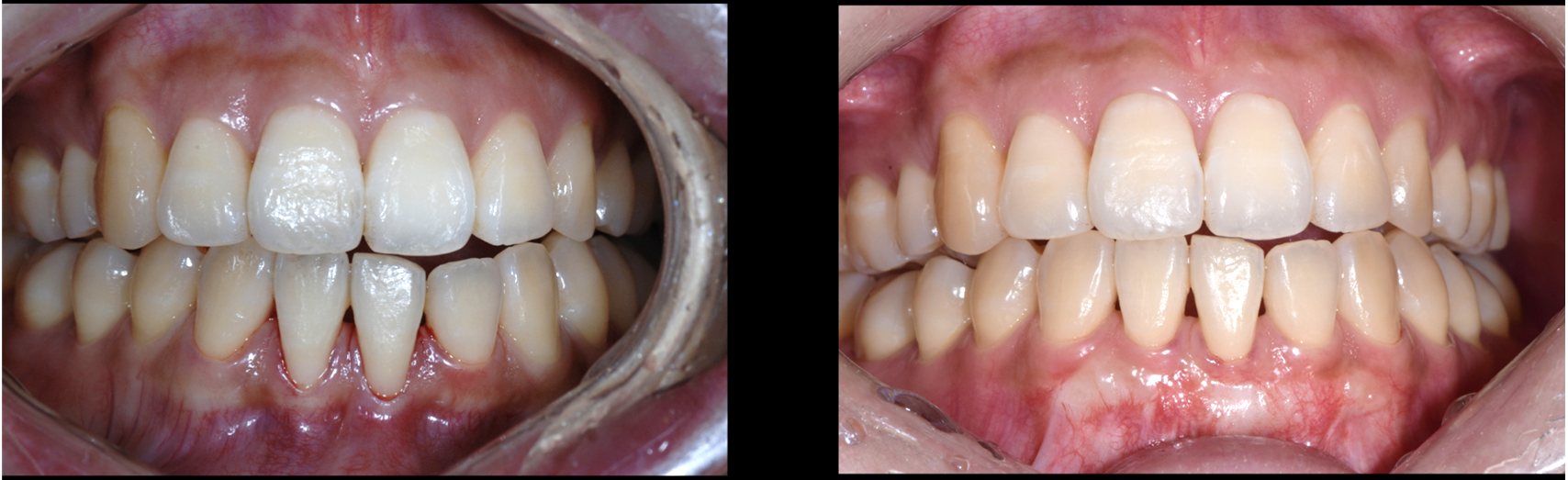

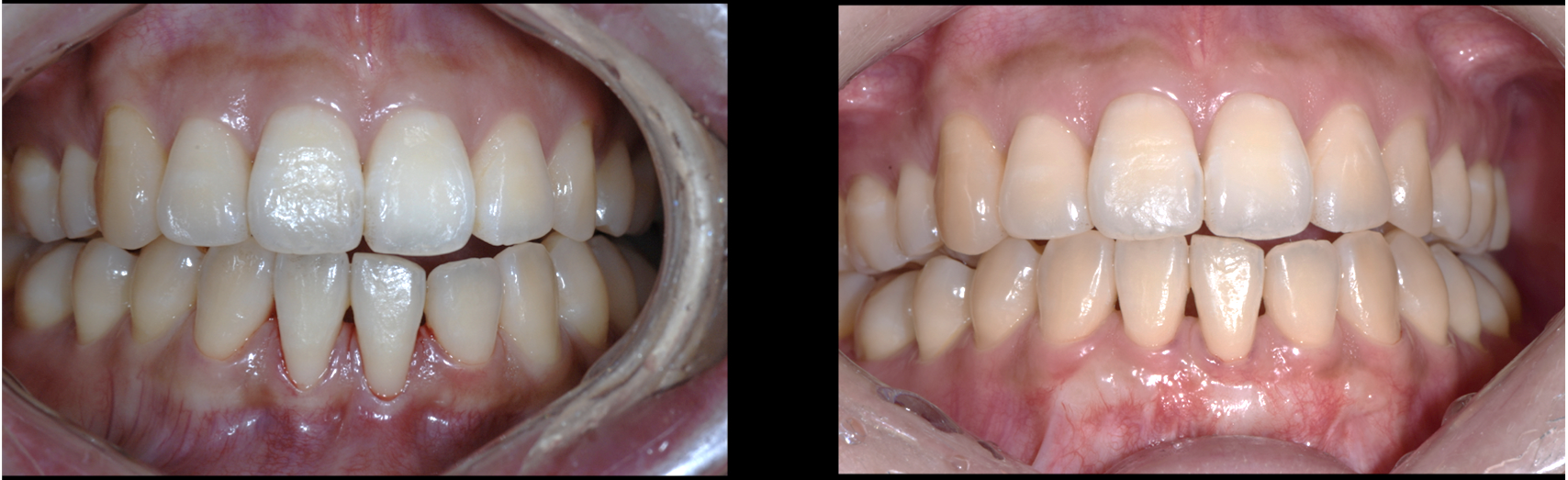


*****

*****

*****

*****

**A**

**B**

**Figure Appendix 1**: **Clinical case of gingival biotype modification with SCTG.** (A) Pre-operation. Gingival recession is observed in the lower anterior teeth (asterisk). The black line is used as a reference line to compare the gingival level before and after the operation. (B) Post-operation. With SCTG, the gingival recession has recovered with an increase in keratinized gingiva.
